# ICT, energy consumption, financial development, and environmental degradation in South Africa

**DOI:** 10.1016/j.heliyon.2021.e07328

**Published:** 2021-06-16

**Authors:** Francis Atsu, Samuel Adams, Joseph Adjei

**Affiliations:** a Business School, Ghana Institute of Management and Public Administration, P.O. Box AH 50, Achimota, Accra, Ghana; b School of Public Service and Governance, Ghana Institute of Management and Public Administration, P.O. Box AH 50, Achimota, Accra, Ghana; c School of Technology, GIMPA, Ghana Institute of Management and Public Administration, P.O. Box AH 50, Achimota, Accra, Ghana

**Keywords:** ICT, Carbon dioxide emissions, Renewable energy, Fossil fuel, Financial development, ARDL

## Abstract

The ICT, energy consumption, and carbon dioxide emissions (CO_2_) relationship is examined for South Africa spanning the period 1970–2019, while controlling for the effects of financial development. The findings of the study based on the Autoregressive Distributed Lag (ARDL), Dynamic Ordinary Least Squares (DOLS), and Fully Modified Ordinary Least Squares (FMOLS) estimators show that ICT and fossil fuel consumption contribute to carbon dioxide emissions, while renewable energy consumption and financial development reduce carbon dioxide emissions. Specifically, the results show that a 1% increase in ICT activities will increase CO_2_ emissions by 0.565% in the long-term, and any temporary shock to this long-run relationship is corrected by 93.20%. Further, there is no evidence of threshold effect of ICT on carbon emissions.

## 1 Introduction

The use of information and communication technology (ICT) tools around the world has increased over the last few decades. Many have described the advances in technology as a prominent feature of the global economy (Danish et al., 2018). It is of interest to note that the gap in technological advancement, particularly in internet connectivity has significantly reduced in the last two decades. For instance, South Africa had internet connectivity of 5.8% in 2000, 24% in 2010 and 56% as at 2016 compared to the sub- Saharan Africa (SSA) average of less 1% in 2000, 7% in 2010 and 24% in 2017. Similarly, mobile phone connectivity is over 150% of the total population, compared to the regional average of 87 per 100 people, making South Africa one of the most highly developed in terms of the ICT infrastructure in the region. The ICTs have many benefits including promoting economic growth and well-being of society (Ziemba, 2019). Recent studies also show that ICTs contribute about 2% of global emissions (Majeed, 2018; Simpson et al., 2019; Ulucak and Khan, 2020).

**Figure 1:**
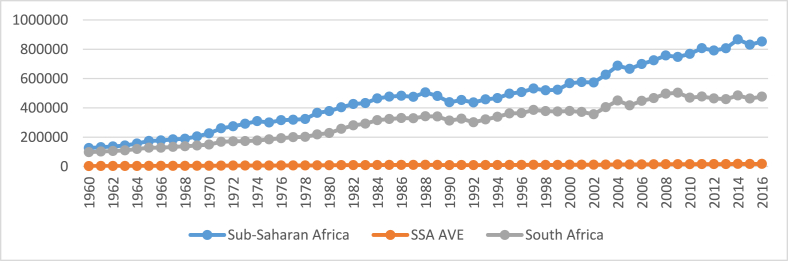
Emissions 1960–2016.

In recent times, ICT, climate change and energy transition have been described as key themes defining human livelihoods. In SSA and South Africa, in particular, climate change is being given serious attention because of its deleterious effect on its economy. Around the globe, climate change associated with the release of greenhouse gases (GHGs) mostly carbon dioxide emissions (CO_2_) is causing serious havoc and could be described as one of the greatest threats to mankind's survival (Landrigan et al., 2018). This is because it is responsible for nearly a sixth of all deaths worldwide and in most severely affected regions, this could be as high as a quarter of deaths (Chersich and Wright, 2019; Johnston, 2019). Though SSA's share of global emissions of CO_2_ is under 3%, over half of this is attributed to South Africa (World Bank, 2019). For example, in the 1960s, it emitted about 77%, declining to 66% in the year 2000, and to 56% in 2016 (See Figure 1). However, the Environmental Performance Index (EPI) shows that South Africa is ranked 4^th^ on the SSA list though around the world it is ranked 95.

The big question is whether the dramatic improvements in ICT is having any effect in mitigating the release of CO_2_ emissions in South Africa? This is the main question to be addressed by the study. Consideration of South Africa is relevant for three main reasons. First, it is the largest economy in SSA and most dependent on fossil fuel (coal) for its energy supply and has the most advanced ICT infrastructure (Kwakwa and Adusah-Poku, 2020; Kreft, Eckstein & Melchior, 2017). Second, South Africa's development is largely dependent on the Agriculture, tourism and forestry sectors which are threatened by high temperatures and rainfall fluctuations associated with climate change (South Africa Department of Environmental Affairs). Indeed, UNEP Report (2020) suggests that South Africa is more likely to be adversely affected by climate change than any other country in SSA because of the very high levels of inequality and poverty. Third, South Africa has the most developed financial sector in the region and therefore we examine whether this has any effect on the ICT, energy consumption and CO_2_ emissions nexus. In achieving the research objective, we control for other factors that are known to affect the evolution of carbon dioxide emissions, particularly, trade and FDI. Finally, we employ both linear and nonlinear estimation techniques to help identify the presence of threshold effects, if any.

For South Africa, in particular, the climate change discussion is seen as a developmental problem and not just an environmental issue (Ziervogel et al., 2014). Thus, an examination of the factors that could help in the mitigation and adaptation of the country to climate change effects is in the right direction. This study contributes to the extant literature in this light. This is situated in the context of the Global Environment Outlook (2019) report that suggests that advances in ICT and technological innovations facilitate the decoupling of economic growth and environmental pollution. As one of the most advanced SSA countries in terms of ICT and technological innovations, the question is how is the advancement in ICTs affecting the environment of South Africa? Is ICT really an enabler that benefits all as noted by Ponelis and Holmner (2015)? It is worth mentioning that, ICT has long been recognized as the backbone of the South African economy (Gilward et al., 2018). Obviously, with welfare losses of nearly US 5 trillion per year attributed to environmental degradation, which is “about 6.2 per cent of global economic output (Landrigan et al., 2018, p. 462), it is obviously an important issue to address, which is the sole purpose of this study.

In the section that follows, we present a brief review of the literature after which the methodology is described, results discussed, conclusions given and policy implications offered.

## 2 Literature review

### 2.1 Theoretical review

There are many theoretical perspectives used to explain the ICT–environmental degradation relationship, including the ecological modernization theory, ICT- led CO_2_ emissions and the Jevons paradox. The ecological modernization theory suggests that ICTs are key determinants of economic growth which usually lead to positive structural change, environmental regulations and technological improvements that reduce environmental pollution (McGranahan, 2010; Poumanyvong and Kaneko, 2010; Sadorsky, 2014; Goundar and Appana, 2018). Thus, ICTs are recognized as a cornerstone of socio-environmental transformation that provides a means of positive change in individual and organizational behavior (Simpson et al., 2019). Bapna et al. (2010) argued from the perspective of the production theory to show that the advantages in ICT come through productivity or efficiency of the production process. Also, the increase in the use of advanced technologies such as internet telephony and video conferencing provide numerous opportunities through online transactions for society at large, which reduces commuting and therefore reduction in CO_2_ emissions. The GeSI's SMARTer 2020 report demonstrates how the increased use of ICT such as video conferencing and smart building management could cut the projected 2020 GHG emissions by 16.5%, amounting to $1.9 trillion in gross energy and fuel savings and a reduction of 9.1 Gigatonnes CO_2_ equivalent (GtCO2e) of greenhouse gases. This is equivalent to more than seven times the ICT sector's emissions in the same period. The increasing awareness of environmental issues and utilization of environmentally friendly technology could also help to reduce environmental pollution. As noted by Nguyen et al. (2019), ICT is related to the creation of cleaner and more ecologically sustainable production process. According to Kouton (2019), the increasing use of e-commerce and e-banking facilitate online transactions which in turn reduce physical travel, thereby reducing greenhouse gas emissions. Other than decreasing dependency on physical travelling, ICT also helps in providing intelligent and automated solutions in various sectors such as power generation, agriculture and manufacturing. As such, ICT is considered as a low carbon enabler and a key determinant of environmental sustainability in various sectors such as power, transportation and buildings. Higon et al. claim that ICT can reduce emissions by building smarter cities, transportation systems, electrical grids and industrial processes. Accordingly, ICT based solutions are expected to help to reduce greenhouse gas emissions basically through the dematerialization. The dematerialization effect of ICT implies a shift from delivering physical products to delivering services.

The ICT- led emissions thesis basically suggests that the design and production of the ICT infrastructure and in many cases their disposal involve emissions that pollute the environment. The GeSI's SMARTer 2020 report and Higon et al. describe ICT's negative effect through what they describe as the “use effect”, which basically refers to the idea that the very design, development, use and disposal of ICT lead to increase in energy consumption which subsequently increase emissions (Shabani and Shahnazi, 2019; Barış-Tüzemen et al., 2020). Presently, the production and use of ICTs contribute about 2% of the global CO_2_ emissions. Zhang and Liu (2015) have argued that energy consumption associated with ICT materials is expected to rise to 430 GW by the end of 2020 from 168GW in 2008. Obviously, this effect is expected to be stronger in the more industrialized countries. It can be inferred that while the ICT- led emissions thesis shows more direct effects, the ecological modernization theory effects are more indirect.

The Jevons paradox or rebound effect unlike the ecological modernization theory does not assume that efficiencies achieved through technological innovations will automatically lead to energy savings or reduction in emissions. Rather, advantages that arise from the energy consumption efficiency which leads to price reduction usually results in increased use of energy services. This “take back” or rebound effect in the energy consumed means a neutral effect of ICT on emissions (Kouton, 2019; Greening et al., 2000, p. 389; Shabani and Shahnazi, 2019). Houghton makes a similar assertion that the more ICT equipment is used, its effect on CO_2_ emissions is likely to dissipate as the increased use leads to increase in energy consumption and therefore increased CO_2_ emissions. The rebound effect is sometimes referred to as the Jevons paradox after Stanley Jevons, (1865) who argued that efficiency renders energy more affordable, which results in the consumption of more energy. Obviously, the idea that efficiency prompts greater energy consumption is indeed paradoxical. Owen (2010) in clarifying this efficiency dilemma, explained that increasing energy efficiency, simply means increasing the productivity of energy, which invariably will lead to price reduction. And because one gets more return for the same money, it stands to reason that demand goes up. The ICT- CO_2_ emissions relationship can therefore not be determined *apriori*. It requires empirical investigation, which is the purpose of this study.

### 2.2 Empirical review

Simpson et al. (2019) investigated the ICT–environment nexus for 113 countries for two decades (1990–2010) and find that the effect of ICT is moderated by the level of development and type of ICT. The findings reveal that fixed telephones lines contributed CO2 emissions in less-developed countries and the internet had similar effects in more developed countries. Similar results were reported by Majeed (2018) in the investigation of the ICT- environment relationship for 132 countries based on the General Method of Moments. Ozcan and Apergis (2018) also studied 20 emerging economies from 1990-2015 and demonstrate that increased ICT access leads to lower levels of air pollution. Nguyen et al. (2020) in a study of the 13 selected G-20 countries using fixed effects and quantile regressions did not find support for the environmental Kuznets Curve, however, they did show that ICT, trade openness and technology contribute to the reduction of emissions. Ahmed and Le. (2020) examine the ICT, trade globalization and carbon emissions dynamics for six Association of Southeast Nations using the panel cointegration techniques and find that both ICT and trade globalization reduce carbon dioxide emissions.

Kunkel and Matthess (2020) study four Sub Sahara African countries and three East Asian and pacific countries and demonstrate that the effect of ICT appears to be country specific among the countries studied. Kwakwa and Adusah-Poku (2020) investigate the industrial structure, urbanization and emissions link for South Africa for a period of 40 years (1975–2014) and find that manufacturing output affects CO_2_ emissions negatively while urbanization had the opposite effect. Mekhum (2020) studied the case of ten Asian countries for the period 1994–2019 and report that application of ICT has a positive effect on environmental quality. Lee and Brahmasrene (2014) investigate nine south eastern nations for the period 1991–2009 and reported that while ICTs facilitate economic growth, they also contribute to environmental degradation. Similar results are reported by Shabani and Shahnazi (2019) for Iran based on Dynamic Ordinary Least Squares estimation technique spanning the period 2002–2013. Batool et al. (2019) studied the South Korean case for over four decades (1973–2016) and show that ICT reduces CO_2_ emissions. Mazzitelli and Aura (2019) study the evolution of CO_2_ emissions in South Africa and find that there is an increasing trend and this is expected to continue in the years ahead. Other studies on South Africa do suggest that improving ICT infrastructure has the potential to improve environmental quality and accordingly there is the need for the country to promote green ICT development (Bok, 2019; Feikie et al., 2017). Similar results are reported by Khan and Ulucak and khan (2020) for the BRICS countries and Latif et al. (2017) for South Asian countries and Chen et al. (2019) and Li et al. (2021) for China.

Kouton (2019) consider the case of 28 SSA countries during the period 2000–2014 using the GMM technique and report that ICT contributes significantly to emissions. However, Ibrahim and Waziri (2020) in a study 45 SSA countries during the period 2008–2016 report that ICT and renewable energy promote environmental quality, while education and trade openness were not significantly related to emissions. In a literature review of the ICT–emissions dynamics, Moletsane (2019) concludes that the benefits of ICT to environmental quality are more likely in developed countries, while the opposite is true for developing countries. Tsauri and Chimbo (2019) introduce interaction terms and report that financial development was a key medium through which ICT contributes to CO_2_ emissions in a study of emerging economies. Equally, Asongu et al. (2017) demonstrates that though ICTs have independent effect on the quality of the environment, they also reduce the negative effect of globalization on environmental quality for SSA countries. Specifically, the authors find that Internet penetration at the national level has a significant negative effect on the CO_2_ emission intensity for all quantiles. A few other studies show non-significant effect of ICT on the environment, including Amri et al. (2018, 2019) and Mbarek and Zghidi (2017) for Tunisia and Salahuddin et al. (2016) for Australia. The review of the literature shows that the effect of ICT on environmental degradation is dependent on country specific characteristics and therefore cannot be determined apriori (Raheem et al., 2020). This study therefore examines the case of South Africa, while accounting for energy consumption, financial development and trade.

The data and methodology employed to achieve the research objectives are discussed next.

## 3 Dataset and econometric model specification

### 3.1 Data

The paper employs time series dataset (sourced from World Development Indictors of the World Bank) to examine the carbon dioxide – ICT (proxied by fixed telephone subscription) of South Africa spanning 1970–2019. For the dependent variable, we use carbon dioxide emissions (mt); while fixed telephone subscription is used as the main independent variable. Following the empirical literature review (Feikie et al., 2017; Asongu et al., 2017; Shabani and Shahnazi, 2019; Simpson et al., 2019; Raheem et al., 2020), we adjust for the effects of the following regressors: (1) renewable energy consumption, (ii) fossil fuel consumption, (iii) trade openness (sum of imports and exports), and (iv) financial development (See Table 1).

**Table 1:** Variable definition and sources.

No	Variable	Definition	Sources
1	*co2*	CO_2_ emissions (metric tons per capita)	WDI
2	*fts*	Fixed telephone subscription	WDI
3	*bmoney*	Broad money (% of GDP)	WDI
4	*dcfs*	Domestic credit provided by financial sector (% of GDP)	WDI
5	*dcps*	Domestic credit to private sector (% of GDP)	WDI
6	*dcbs*	Domestic credit to private sector by banks (% of GDP)	WDI
7	*fdi*	Foreign direct investment, net inflows (% of GDP)	WDI
6	*gdp*	Gross domestic product (GDP per capita)	WDI
7	*trade*	Trade (sum of exports and imports) (% of GDP)	WDI

Notes: The term WDI represents the World Bank's world development indicators database.

### 3.2 Econometric model specification

The paper employs a model specification that draws various variables from literature (Asongu et al., 2017; Mazzitelli and Aura, 2019; Shabani and Shahnazi, 2019; Barış-Tüzemen et al., 2020; Kunkel and Matthess, 2020; Kwakwa and Adusah-Poku, 2020) presented below:

(1) $$\ln\;co2_{t}=\beta_{1}+\beta_{2}\;\ln\;fts_{t}+\beta_{3}\;\ln\;ren_{t}+\beta_{4}\;\ln\;foss_{t}+\beta_{5}\;\ln\;trade_{t}+\beta_{6}\;\ln\;fin_{t}+\beta_{7}\;\ln\;gdp_{t}+\varepsilon_{t}$$

where CO_2_ is carbon dioxide emissions, fts is information and communication technology proxied by fixed telephone subscription, ren renewable energy consumption, foss is fossil fuel consumption, trade is trade openness, fin is financial development variable, gdp is GDP per capita and ε is the residual term. The terms $\beta_{0},\cdots ,\beta_{7}$ are the respective coefficients of the above variables to be estimated.

For the sake of parsimony, we reparametrize Eq. (1) in the spirit of Pesaran et al.‘s (2001) ARDL technique which is appropriate for estimating coefficients using a mixture of regressors of 0 and 1 orders of integration. The ARDL “bounds test” using F test is used to determine the existence of a long-run associations of the covariates and the coefficients are then estimated. Hence, we employ the standard ARDL (p,q) model and state our model as follows:

(2) $$\begin{array}{l} \Delta \;\ln\;co2_{t}=\alpha_{0}+\gamma_{1}\;\ln\;co2_{t-1}+\gamma_{2}+\ln\;fts_{t-1}+\gamma_{3}\;\ln\;ren_{t-1}+\gamma_{4}\;\ln\;foss_{t-1}\\ \;+\gamma_{5}\;\ln\;trade_{t-1}+\gamma_{6}\;\ln\;fin_{t-1}+\gamma_{7}\;\ln\;gdp_{t-1}+\overset{p}{\underset{i=1}{\sum }}\beta_{1i}\Delta \;\ln\;co2_{t-i}+\overset{q}{\underset{i=0}{\sum }}\beta_{2i}\Delta \;\ln\;fts_{t-i}\\ \;+\overset{q}{\underset{i=0}{\sum }}\beta_{3i}\Delta \;\ln\;ren_{t-i}+\overset{q}{\underset{i=0}{\sum }}\beta_{4i}\Delta \;\ln\;foss_{t-i}+\overset{q}{\underset{i=0}{\sum }}\beta_{5i}\Delta \;\ln\;trade_{t-i}+\overset{q}{\underset{i=0}{\sum }}\beta_{6i}\Delta \;\ln\;fin_{t-1}\\ \;+\overset{q}{\underset{i=0}{\sum }}\beta_{7i}\Delta \;\ln\;gdp_{t-1}+\varepsilon_{t} \end{array}$$

where Δ represents the first difference operator, *ln* is the natural log operator, $\alpha_{0}$ is a constant, $\gamma_{1},\cdots ,\gamma_{7}$ denote the coefficients of the lagged levels of the dependent and independent variables, $\beta_{1},\cdots ,\beta_{7}$ are the coefficients of the differenced lagged regressors. Further, the terms *p* and *q* are the optimal lag lengths, and $\varepsilon_{t}$ represents the error term.

## 4 Empirical analysis

### 4.1 Summary statistics and unit root tests

Panel A and Panel B of Table 2 respectively present the descriptive statistics and pairwise correlation among the variables under study. The variables *fts* and *gdp* have the lowest and highest averages of 7.908 and 3681.103, whilst the averages of the other variables fall within the forgoing range. *foss* exhibits the least dispersion as compared to *gdp* that shows the most dispersion in their values. In exception of *ren* and *fin*, all the variables are negatively skewed. All the regressors show negative excess kurtosis. The pair-correlation matrix shows that there are no high correlations among variables.

**Table 2:** Descriptive statistics and correlation.

Panel A: Descriptive Statistics							
Statistic	*co2*	*fts*	*ren*	*foss*	*fin*	*trade*	*gdp*
Mean	8.593	7.908	17.278	87.364	86.676	52.791	3681.103
Median	8.598	8.301	17.094	87.135	84.475	52.730	3146.993
Max.	9.979	12.388	19.121	90.506	127.541	72.865	8007.413
Mini.	6.786	3.458	15.570	84.243	53.358	37.487	834.553
Std. Dev.	0.838	2.354	0.817	1.664	23.899	7.674	1946.299
Skewness	-0.126	-0.213	0.176	0.013	0.240	0.046	0.523
Kurtosis	2.211	1.895	2.307	2.009	1.456	2.667	2.181
JB	1.431	2.923	1.259	2.047	5.447	0.249	3.673
Prob.	0.489	0.232	0.533	0.359	0.066	0.883	0.159
Obs.	50	50	50	50	50	50	50
**Panel B: Pairwise Correlation**							
*co2*	1.000						
*fts*	0.467	1.000					
*ren*	-0.669	-0.088	1.000				
*foss*	-0.109	-0.746	-0.142	1.000			
*fin*	0.310	0.602	-0.316	-0.508	1.000		
*trade*	0.141	-0.093	-0.415	0.122	0.498	1.000	
*gdp*	0.450	0.400	-0.518	-0.370	0.821	0.519	1.000

*Notes*: The term *fin* is the composite index of financial development obtained from the individual measures of financial development (*dcps, dcbs, dcfs*, and *bmoney*) using Cronbach's Alpha method as presented in Table 3.

Following literature, we perform item-analysis in Table 3 for the financial development index using the Cronbachs Alpha technique. The following individual measures of financial development are used: (i) domestic credit to private sector, (ii) domestic credit to private sector by banks, (iii) domestic credit provided by the financial sector, and (iv) broad money. The test scale shows a reliability value of 0.852 which is appropriate since it is above the acceptable range of 0.7–0.8.

**Table 3:** Item-analysis of financial development measures.

Item	Obs	Sign	Item-test correlation	Item-rest correlation	alpha
*dcfs*	48	+	0.995	0.984	0.705
*dcps*	48	+	0.992	0.980	0.662
*dcbs*	49	+	0.939	0.926	0.849
*bmoney*	50	+	0.720	0.657	0.882
Test scale (fin)					0.852

Notes: Using the STATA “alpha”, we compute the Cronbach's Alpha where, per literature, values within the range 0.7 and above are more appropriate.

To proceed with the ARDL methodology, we examine the stationarity of the variables using Augmented Dickey-Fuller and Phillips-Perron unit root tests in Table 4a. Specifically, Panel A offers unit root tests in levels, while Panel B offers the tests in first differences. All the variables are integrated of order 1 (I (1)).

**Table 4a:** Augmented Dickey-Fuller (ADF) and Phillips-Perron (PP) unit root tests.

Variable	ADF-Test		PP-test		Outcome
	Const	Const + Trend	Const	Const + Trend	
**Panel A: Levels**					
lnco2	-2.655∗	-2.314	-2.711∗	-2.435	Non-stationary
lnfts	0.074	-2.244	-0.563	-1.056	Non-stationary
lnren	-2.867∗	-2.903	-2.570	-2.766	Non-stationary
lnfoss	-1.631	-1.684	-1.616	-1.748	Non-stationary
lntrade	-1.894	-2.083	-1.844	-2.061	Non-stationary
lnfin	-1.440	-1.332	-1.440	-1.044	Non-stationary
lngdp	-2.345	-3.220∗	-2.090	-2.651	Non-stationary
**Panel B: First differenced**					
lnco2	-7.255∗∗∗	-7.264∗∗∗	-7.234∗∗∗	-7.246∗∗∗	I(1)
lnfts	-7.526∗∗∗	-7.670∗∗∗	-5728∗∗∗	-6.029∗∗∗	I (1)
lnren	-4.884∗∗∗	-4.828∗∗∗	-6.911∗∗∗	-6.838∗∗∗	I (1)
lnfoss	-7.854∗∗∗	-7.822∗∗∗	-7.796∗∗∗	-7.781∗∗∗	I (1)
lntrade	-6.350∗∗∗	-6.280∗∗∗	-6.551∗∗∗	-6.458∗∗∗	I (1)
lnfin	-6.988∗∗∗	-6.968∗∗∗	-6.988∗∗∗	-6.968∗∗∗	I (1)
lngdp	-5.380∗∗∗	-5.603∗∗∗	-4.763∗∗∗	-4.757∗∗∗	I (1)

The study further investigates whether the variables remain a combination of I (0) and I (1) time series if structural breaks are present using the Zivot-Andrews (ZA) and Narayan and Popp (2010, 2013) tests. The outcomes presented in Tables 4b and 4c show that all the variables are I (1) series except lnfoss which is an I(0) series when there are two structural breaks in the intercept and the slope.^1^

Therefore, from all the unit root tests, we confirm that our variables are a combination of I (0) and I (1) as required by Pesaran et al. (2001).

**Table 4b:** Zivot Andrews Unit root test.

Variable	Levels				First Difference		Outcome
	Intercept		Intercept + trend		Intercept	Intercept + trend	
	t-Stat.	Break date	t-Stat.	Break date	t-Stat.	t-Stat.	
*lnco2*	-3.378	1979	-3.402	1992	-7.707∗∗∗	-7.901∗∗∗	I(1)
*lnfts*	2.449	2012	-0.807	1998	-3.276∗∗	-4.087∗∗∗	I(1)
*lnren*	-3.685∗∗	1991	-3.839∗∗	1991	-5.437∗∗∗	-5.658∗∗∗	I(1)
*lnfoss*	-3.881∗∗∗	2003	-3.702∗∗	1998			I(0)
*lnfin*	-2.929	1992	-2.554	2011	-8.171∗∗∗	-8.527∗∗	I(1)
*lntrade*	-3.412	1982	-3.432	1989	-6.539∗∗∗	-6.754∗∗∗	I(1)
*lngdp*	-3.551	1998	-3.956	2010	-5.520∗∗∗	-5.990∗∗∗	I(1)

Notes: Intercept denotes unit root with a structural break in the intercept only, while Intercept + trend denotes root unit with a structural break in the intercept and trend. ∗∗∗/∗∗/∗ represent significance at 1%/5%/10%, respectively.

**Table 4c:** Results for Narayan and Popp (2010, 2013) unit root test with two structural breaks.

Panel A: Levels						
Variable	Break in intercept only			Break in intercept and slope		
	t-Stat.	TB1	TB2	t-Stat.	TB1	TB2
*lnco2*	-2.231	2002	2004	-3.327∗∗	1989	2003
*lnfts*	0.903	1986	1999	1.496	1996	1999
*lnren*	-2.270	1998	2009	-2.796	1990	2009
*lnfoss*	-1.647	1988	1992	-4.242∗∗∗	1987	2003
*lnfin*	-0.454	1991	2001	-0.693	1991	2001
*lntrade*	-2.006	2002	2008	-1.351	1982	2008
*lngdp*	-3.006	1979	2002	-3.027∗	1979	2002

Panel B: First Difference							Outcome
Variable	Break in intercept only			Break in intercept and slope			
	t-Stat.	TB1	TB2	t-Stat	TB1	TB2	
*lnco2*	-7.208∗∗∗	1989	2002	-6.394∗∗∗	2002	2004	I (1)
*lnfts*	-8.936∗∗∗	1999	2001	-8.908∗∗∗	1999	2001	I (1)
*lnren*	-5.438∗∗∗	1998	2009	-5.443∗∗∗	2001	2009	I (1)
*lnfoss*	-7.905∗∗∗	1992	2000				I (0)/I (1)
*lnfin*	-7.261∗∗∗	1991	2001	-7.902∗∗∗	1991	2001	I (1)
*lntrade*	-6.338∗∗∗	2002	2008	-6.300∗∗∗	2002	2008	I (1)
*lngdp*	-6.347∗∗∗	1983	2002	-6.972∗∗∗	1983	2002	I (1)

*Notes*: TB1 and TB2 represent the two break dates. ∗∗∗/∗∗/∗ significant at 1%, 5% and 10%, respectively.

### 4.2 Regression output discussions

We present discussions of the empirical outputs of two models (i) basic model that explores the impact of fixed telephone subscription while adjusting for the effect of renewable energy consumption and fossil fuel consumption, and (ii) standard model where the basic model is controlled for the effect of trade openness, financial development and GDP per capita.^2^ The diagnostics and estimates of Model 1 are presented in Panel A and Panel B of Table 5, respectively. The F-statistics of the Bounds test is greater than the critical value of the upper bound suggesting that there is a long-run relationship between the variables based on ARDL (1,1,1) specification. We explore the diagnostics of the model as follows: (i) serial correlation is studied using Breusch-Pagan-Godfrey LM serial correlation where the null hypothesis is no serial correlation, (ii) heteroscedasticity is examined by employing ARCH and Breusch-Godfrey tests where the null hypotheses of homoscedasticity or constant variance, and (iii) specification errors (e.g. omitted variables, incorrect functional form) are examined using Ramsey RESET test where the null hypothesis of correct specification. The null hypotheses of all the tests are maintained suggesting that our models have passed all the tests. In the short-run, fixed telephone subscription (lnfts) contributes to carbon dioxide emissions implying that 1% increase in fixed telephone subscription will boost emission by 0.255%. This outcome is consistent with the study of Shabani and Shahnazi (2019) for Iran, Mazzitelli and Aura (2019) for South Africa, but does not support the study of Asongu et al. (2017) for 44 SSA countries, Ulucak and Khan (2020) for the BRICS region. Renewable energy consumption reduces carbon emissions, while fossil fuel consumption contributes to carbon emissions which supports the study of Khan et al. for China, Mensah et al. (2019) and Churchill et al. (2018) for OECD countries, and Wang and Zhu (2020) for China. A temporary shock to the long-run equilibrium is corrected by 60.6%. In the long-run, all the variables maintained their respective significant impacts. The regression diagnostics and coefficients of Model 2 are respectively presented in Panel A and Panel B of Table 6. The F-statistic (4.242) of the specification ARDL (1, 4, 3, 3, 1, 4, 4) is more than the upper critical value at 1% significance. Hence, there exists a long-run relationship among the variables, and any short-term shock to this relationship is corrected by 93.2%. Except serial correlation test at 1% significance, Model 2 has passed all the diagnostic tests. Although, there are some lag effects of the regressors on carbon emissions, there are no short-run contemporaneous effects of the regressors on the carbon emissions. For the long-run output, however, fixed telephone subscription promotes emissions. For instance, a 1% increase in fixed telephone subscription promotes emissions by 0.565%. Fossil fuel consumption and trade openness drive carbon emissions by 5.843% and 0.283%, respectively. Financial development dampens carbons by 0.321% when it is increased by 1%. However, renewable energy and GDP per capita do not contribute to emissions.

**Table 5:** Long-run relationship and Estimates of ARDL specification.

Panel A: Test for Long-run relationship				
Model 1- Bounds F-test				
ARDL specification	F-statistic	SL	I(0)	I(1)
ARDL (1,1,1)	5.488∗∗∗	10%	2.370	3.200
		5%	2.790	3.670
		1%	3.650	4.660
**Residualdiagnostics and function form**	**stat**	**p-value**		
Ramsey RESET	1.616	0.115		
Breusch-Godfrey Serial Correlation LM	0.151	0.860		
Heteroskedasticity Breusch-Pagan-Godfrey	0.609	0.745		
ARCH Heteroskedasticity	0.169	0.683		

Panel B: Parameter Estimates of the ARDL model					
Model 1	Variable	Coef.	Std. Error	t-Stat	Prob.
Short-run	Δlnfts	0.255∗∗	0.100	2.560	0.015
	Δlnren	-0.429∗∗	0.180	-2.389	0.022
	Δlnfoss	1.684∗∗	0.716	2.352	0.024
	ect(t-1)∗	-0.606∗∗∗	0.110	-5.507	0.000
Long-run	lnfts	0.347∗∗∗	0.071	4.902	0.000
	lnren	-0.768∗∗∗	0.277	-2.776	0.009
	lnfoss	4.349∗∗∗	1.206	3.605	0.001
	constant	-15.809	6.050	-2.613	0.013

Notes: The optimal lag lengths are determined by SBIC and Δ is the difference operator. The standard errors are in the parenthesis, and ∗∗∗/∗∗/∗ denote 1%,5% and 10% significance, respectively. The term ect denotes the error correction term.

**Table 6:** Long-run relationship and estimates of ARDL specification.

Panel A: Test for Long-run relationship				
Model 1- Bounds F-test				
ARDL specification	F-statistic	SL	I(0)	I(1)
ARDL (1, 4, 3, 3, 1, 4, 4)	4.242∗∗∗	10%	1.990	2.940
		5%	2.270	3.280
		1%	2.880	3.990
**Residualdiagnostics and function form**	**stat**	**p-value**		
Ramsey RESET	0.533	0.602		
Breusch-Godfrey Serial Correlation LM	12.804	0.010		
Heteroskedasticity Breusch-Pagan-Godfrey	0.680	0.814		
ARCH Heteroskedasticity	0.035	0.853		

Panel B: Parameter Estimates of the ARDL model					
Model 2	Variable	Coef.	Std. Error	t-Stat	Prob.
Short-run	Δlnfts	0.125	0.103	1.207	0.245
	Δlnfts(t-1)	-0.368∗∗∗	0.124	-2.962	0.009
	Δlnfts(t-2)	0.018	0.148	0.121	0.906
	Δlnfts(t-3)	-0.595∗∗∗	0.130	-4.575	0.000
	Δlnren	0.347	0.224	1.549	0.141
	Δlnren(t-1)	-0.494∗∗	0.196	-2.519	0.023
	Δlnren(t-2)	-0.556∗∗	0.200	-2.779	0.013
	Δlnfoss	-0.418	0.762	-0.548	0.591
	Δlnfoss(t-1)	-3.851∗∗∗	1.014	-3.796	0.002
	Δlnfoss(t-2)	-2.163∗∗	0.880	-2.457	0.026
	Δlntrade	-0.124	0.071	-1.731	0.103
	Δlnfin	0.010	0.063	0.167	0.870
	Δlnfin(t-1)	0.464∗∗∗	0.093	5.006	0.000
	Δlnfin(t-2)	0.284∗∗∗	0.076	3.743	0.002
	Δlnfin(t-3)	0.170∗∗	0.063	2.677	0.017
	Δlngdp	-0.086∗	0.044	-1.959	0.068
	Δlngdp(t-1)	0.084	0.052	1.619	0.125
	Δlngdp(t-2)	-0.073	0.048	-1.508	0.151
	Δlngdp(t-3)	-0.083∗	0.046	-1.809	0.089
	ect(t-1)∗	-0.932∗∗∗	0.133	-6.984	0.000
Long-run	lnfts	0.565∗∗∗	0.129	4.381	0.001
	lnren	0.211	0.593	0.356	0.726
	lnfoss	5.843∗∗∗	1.484	3.937	0.001
	lntrade	0.283∗	0.149	1.901	0.075
	lnfin	-0.321∗∗	0.132	-2.429	0.027
	lngdp	0.045	0.040	1.136	0.273
	constant	-25.787	8.391	-3.073	0.007

*Notes*: The optimal lag lengths are determined by SBIC and Δ is the difference operator. The standard errors are in the parenthesis, and ∗∗∗/∗∗/∗ denote 1%,5% and 10% significance, respectively. The term ect denotes the error correction term.

**Figure 2:**
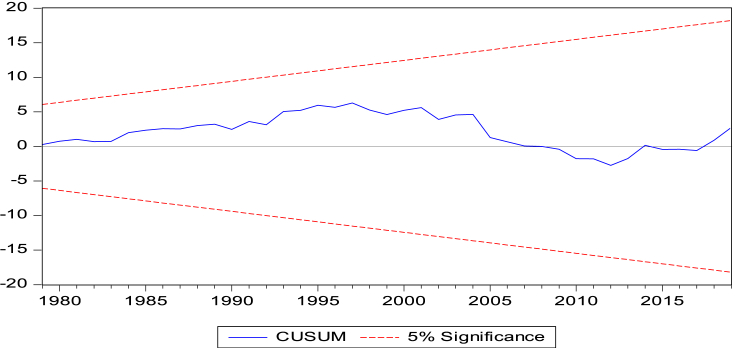
Cumulative Sum of Recursive Residuals (CUSUM) Plot. Notes: Lines depict critical bounds at 5% level of significance.

**Figure 3:**
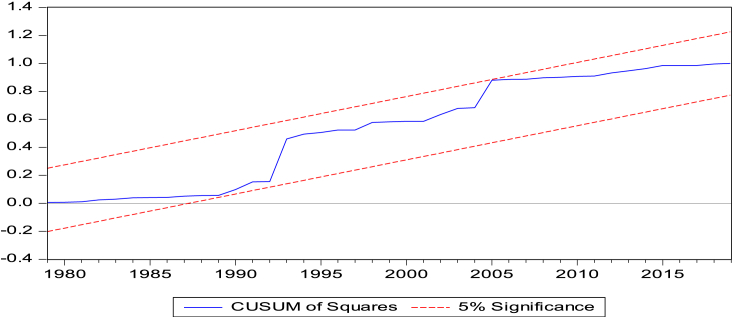
Cumulative Sum of square of Recursive Residuals (CUSUMSQ) Plot. Notes: Lines depict critical bounds at 5% level of significance.

The long-run model shows the following results. Fixed telephone subscription, fossil fuel consumption and trade openness increase carbon emissions, while financial development dampens emissions by 0.321% if it is improved by 1%. The tests-cumulative sum of recursive (CUSUM) and cumulative sum of squares of recursive (CUSUMSQ) in Figures 2 and 3, respectively, reveal that our models are stable over time since the plots fall within the critical bounds of 5% significance.

### 4.3 Robustness check

For robustness checks, we follow Apergis (2016), Nguyen et al. (2020), Samargandi (2019), among others, and re-estimate Model 1 and Model 2 using Fully-modified OLS (FMOLS) and Dynamic OLS (DOLS) estimators and the results are respectively presented in Panel A and Panel B of Table 7. Comparing the results with the corresponding long-run coefficients in Tables 5 and 6, the empirical analysis shows the following. The FMOLS estimates of Model 1 are similar to those of ARDL for Model 1. Specifically, fixed telephone subscription and fossil fuel consumption positively drive carbon emissions as compared to the renewable energy consumption that dampens carbon emissions. For Model 2, fixed telephone subscription, fossil fuel consumption and GDP per capita promote carbon emissions, while renewable energy consumption and financial development inhibit carbon emissions. Trade openness has no significant impact on carbon emissions. The results for Model 2 using FMOLS are similar to those of ARDL, except the marginal variations in the impact of GDP per capita and renewable energy on carbon emissions. Further, the parameter estimates of DOLS are similar to those of FMOLS except for economic growth which is insignificant for the DOLS, and financial development, which is significant at 1% for FOMLS but significant at 5% for DOLS.

**Table 7:** Fully-modified OLS and Dynamic OLS regression estimates.

Variable	Model 1			Model 2		
	Coef.	Std. Error	Prob.	Coef.	Std. Error	Prob.
**Panel A: Fully-modified Ordinary Least Squares (FMOLS) Estimates**						
lnfts	0.194∗∗∗	0.043	0.000	0.185∗∗∗	0.025	0.000
lnfoss	1.448∗	0.732	0.054	1.240∗∗∗	0.402	0.004
lnren	-1.160∗∗	0.214	0.000	-0.964∗∗∗	0.144	0.000
lnfin				-0.136∗∗∗	0.034	0.000
lntrade				-0.025	0.046	0.592
lngdp				0.070∗∗∗	0.016	0.000
Constant	-1.411	3.599	0.697	-0.883	2.080	0.674
**Panel B: Dynamic Ordinary Least Squares (DOLS) Estimates**						
lnfts	0.402∗∗∗	0.059	0.000	0.338∗∗∗	0.051	0.000
lnfoss	4.514∗∗∗	0.987	0.000	2.792∗∗∗	0.705	0.000
lnren	-0.671∗∗	0.260	0.015	-0.862∗∗∗	0.189	0.000
lnfin				-0.108∗∗	0.048	0.028
lntrade				0.011	0.064	0.863
lngdp				0.021	0.025	0.394
Constant	-16.947	5.111	0.002	-8.304	3.488	0.022

*Notes*: We employed Kao (Engle-Granger based) and Fisher (Combined Johansen) panel cointegration technique to examine the cointegration relationship among the variables under study. The standard errors are in the parenthesis, and ∗∗∗/∗∗/∗ denote 1%,5% and 10% significance, respectively.

## 5 Conclusion and policy implications

The ICT, energy consumption, and carbon dioxide emissions (CO_2_) relationship is examined for South Africa spanning the period 1970–2019, while controlling for the effects of financial development as the country with the most developed financial sector in sub-Sahara Africa. The findings of the study based on the ARDL, DOLS and FMOLS analytical tools and framework show that ICT and fossil fuel consumption contribute to or worsen carbon dioxide emissions, while renewable energy consumption and financial development dampen carbon dioxide emissions. Specifically, the results show that a 1% increase in ICT activities will increase CO_2_ emissions by 0.565% in the long-term, and any temporary shock to this long-run relationship is corrected by 93.20%. Further, we did not find any evidence of a non-linear relationship between ICT and carbon emissions. Three main policy implications can be drawn from the findings of the study.

First, the transition to clean energy has to be taken seriously especially in the era of capital constraints for many countries in Africa. The implication is that the government of South Africa should focus on deepening institutional quality and regulatory framework for both energy policy targeted at increasing the use of renewables in the electricity generation mix. Studies on South Africa discussed earlier that improving institutional quality helps to improve environmental quality. Additionally, in support of the findings of this study, the reported studies show that while fossil fuel consumption contributes to environmental degradation, the transition to clean energy promotes environmental quality. These findings show that South Africa will need to intensify the diversification of energy sources to include more renewables and other clean energy sources and reduce its dependence on coal. Currently, more than 90% of South Africa's production is from coal and thus is one of top ten countries in the export and consumption of coal and other fossil fuels, which contribute greatly to environmental degradation. It is worthy of mention that the South African government has recognized the adverse effects of coal on its environment and in the last two decades has taken action to reduce its dependence. The strategic intent to increase renewables actually began in 2003 and buoyed by initiatives like the Integrated Resource Plan (IRP) and Renewable Energy Independent Power Producer Procurement Programme (REIPPPP), it is expected that generation of electricity from renewables (for example, solar and biomass), could help reduce environmental pollution. From the policy perspective, South Africa should also seek to promote nuclear energy in its energy mix to improve environmental quality. South Africa's commitment to diversification of its energy resources is well recognized, as it is the only country in sub-Sahara Africa with a functional nuclear power for electricity which is about 5% of its generating capacity.

Second, the findings show that though ICT has costs, overall, it helps to improve environmental quality. This indicates that South Africa should continue to update and upgrade its ICT infrastructure to gain the twin benefits of increased economic output and reduced CO_2_ emissions. Obviously, with an ICT infrastructure that is one of the most advanced in the region, the policy space should be created to leverage ICT in the strategy to reduce environmental degradation. It is worthy of note that in South Africa and many of the SSA countries, ICT infrastructure (internet, broadband, and mobile phones) has increased significantly and therefore there is the need to identify how best they could be deployed to reduce emissions that are detrimental to the environment. The government of South Africa should examine the consumption, production, and technological innovations that ICT brings and also ensure that the research and development and technological innovations far outweigh the consumption and production benefits to reduce environmental pollution. While the consumption and production benefits lead to increase in the quality of life and therefore utilization of machinery and equipment that release emissions, the research and development should help to produce more low-carbon machinery to reduce CO_2_ emissions. As reported earlier, though ICTs help to improve the well-being of society, it also contributes to global emissions through use and release of waste especially from old equipment. Accordingly, it is recommended that policies should be promulgated to guide what kinds of products that could be imported and in certain cases discouraged or banned.

Third, improvements in financial development are important in reducing environmental degradation. This suggests that as the highest emitter of CO_2_ emissions in the SSA region, development of its financial sector could be a critical means to mitigate climate change effects. This is attributed to the fact that increasing the level of financial depth serves as a channel or a driver for development of technological and innovative products and services to enhance energy efficiency. Indeed, many studies do suggest that promoting financial development is beneficial to firms because it removes credit constraints and helps to increase investment in advance technology that is environmentally friendly. As mentioned earlier, the transition to clean energy requires financing and therefore the development of financial institutions and instruments are in the right direction to help reduce environmental degradation. More practically, the South African government should also implement policies that are conducive in attracting foreign direct investment and more particularly invest in research and development critical in promoting green development- thus promoting growth that does not occur at the expense of the environment. The findings of the study are limited by the fact that it employs only fixed telephone lines due to data constraints. Future research should look at the effect of other ICT tools (internet, mobile phones, and broadband) as data becomes available. Additionally, more cross-country studies should be conducted to provide policy directions for the African region as a whole in its desire to promote economic growth and environmental quality.

## Declarations

### Author contribution statement

Francis Atsu: Conceived and designed the experiments; Performed the experiments; Analyzed and interpreted the data; Contributed reagents, materials, analysis tools or data; Wrote the paper.

Samuel Adams: Conceived and designed the experiments; Performed the experiments; Contributed reagents, materials, analysis tools or data; Wrote the paper.

Joseph Adjei: Performed the experiments; Contributed reagents, materials, analysis tools or data; Wrote the paper.

### Funding statement

This research did not receive any specific grant from funding agencies in the public, commercial, or not-for-profit sectors.

### Data availability statement

Data will be made available on request.

### Declaration of interests statement

The authors declare no conflict of interest.

### Additional information

No additional information is available for this paper.

## Footnotes

1 The Narayan and Popp (2010) considers two different specifications: specification 1 adjusts for two structural breaks in the level of the series, while specification 2 accounts for two structural breaks in the level and slope of the series.

2 We explored for the existence of threshold effects and found no evidence. However, for the sake brevity, the results are not presented here but available upon request.
